# Supplementary material for: MitoScape: A big-data, machine-learning platform for obtaining mitochondrial DNA from next-generation sequencing data
Source: PLoS Comput Biol. 2021 Nov 11;17(11):e1009594. doi: 10.1371/journal.pcbi.1009594 (PMC8610268; doi:10.1371/journal.pcbi.1009594)
Supplement: S1 Supplementary Methods — (DOCX) [file pcbi.1009594.s001.docx]

## S1 SUPPLEMENTARY METHODS

## Human mtDNA primers:

FWD2: 5’ CAT CAA CAA CCG ACT AAT CAC C 3’

8637-8658

REV2: 5’ CGC AAT GCT ATC GCG TGC ATA C 3’

92-71

FWD7_BL: 5’ GTG AAC TGT ATC CGA CAT CTG G 3’

16483-16504

REV7_BL: 5’ AGT GTA GAG GGA AGG TTA ATG G 3’

 9095-9075

## Final Protocols:

Prepared using the Qiagen LongRange PCR kit:

**Note:** All reagents thawed on ice and mixed using benchtop vortex before use.

1. A reaction mix was prepared as per the following table in a 1.5ml Eppendorf tube, mixed thoroughly with a benchtop vortex and touch centrifuged.

| **Reagent** | **Volume/reaction (μL)** |
| --- | --- |
| LR-PCR Buffer (10x) | 5 |
| dNTP (10mM) | 2.5 |
| FWD Primer (10μM) | 2 |
| REV Primer (10μM) | 2 |
| RNase-free H_2_O | 32 |
| LR-PCR Enzyme Mix | 0.4 |
| **Total** | **43.9** |

1. 43.9μL of the master mix was added to each PCR tube
2. 100ng of DNA (determined by NanoDrop) was added to each PCR tube, and RNase-free water added to bring total volume to 50μL/tube.
3. Tubes were touch centrifuged twice, separated by thorough mixing.
4. PCR was performed by thermal cycler under the following conditions

**For FWD/REV2**

|  | x 35 | | |  |
| --- | --- | --- | --- | --- |
| Initial Activation | Denaturing | Annealing | Extension | End |
| 3:00 | 0:15 | 0:30 | 8:00 | ∞ |
| 93^o^C | 93 ^o^C | 62 ^o^C | 68 ^o^C | 4 ^o^C |

**For FWD/REV7_BL**

|  | x 35 | | |  |
| --- | --- | --- | --- | --- |
| Initial Activation | Denaturing | Annealing | Extension | End |
| 3:00 | 0:15 | 0:30 | 8:00 | ∞ |
| 93^o^C | 93 ^o^C | 59 ^o^C | 68 ^o^C | 4 ^o^C |

1. PCR products were visualized using electrophoresis on a 0.7% agarose gel with 10μL of GelRed/100ml TAE. The gel was run at 40V for 3hrs.

FWD/REV2


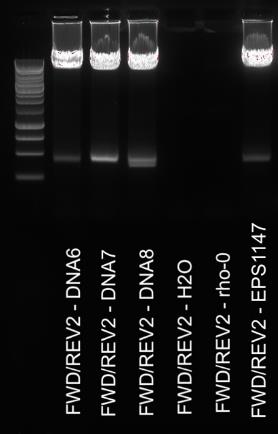


Four human DNA samples show robust amplification.

Lack of amplification in rho-zero cell DNA lane.

* this faint spurious band appeared at annealing temp of
 59 C. Raising this temp to 62 C as per the protocol
 eliminated this band.

FWD/REV7_BL


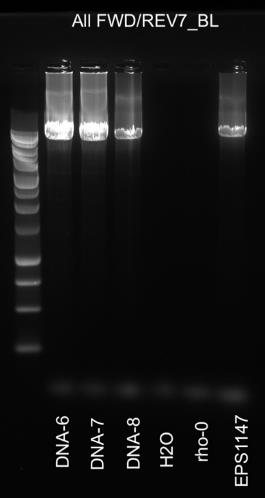


**BLAST:**

The primers were checked for undesirable matches to chromosomal DNA using NCBI’s BLAST. FWD2 was shown to have a 100% match to a region on Chromosome 10; all other primers had no 100% matches to chromosomal DNA. BLAST check for NUMTs – Exact sequence matches shown:

**FWD2:**


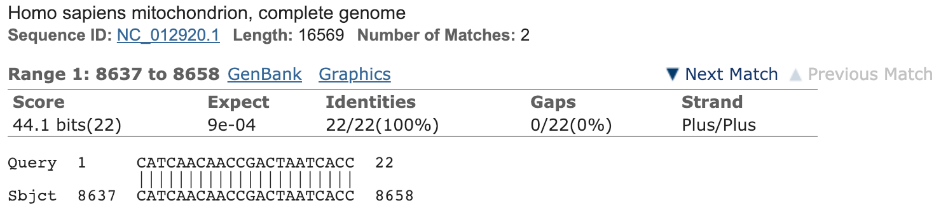


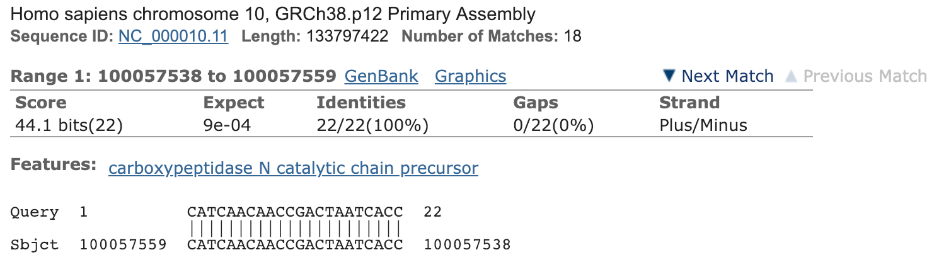


**REV2:**


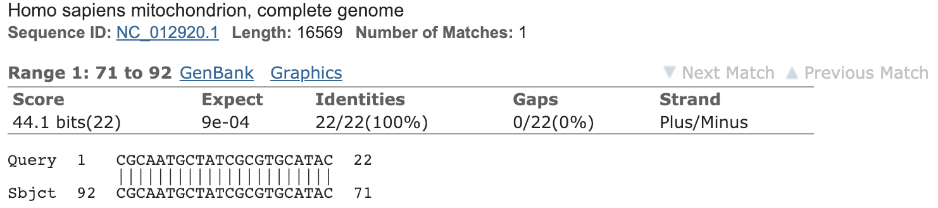


**FWD7_BL:**


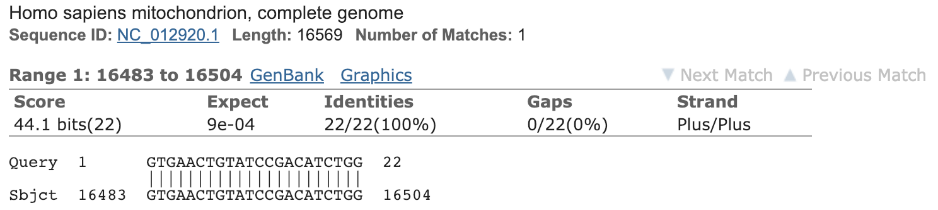


**REV7_BL:**


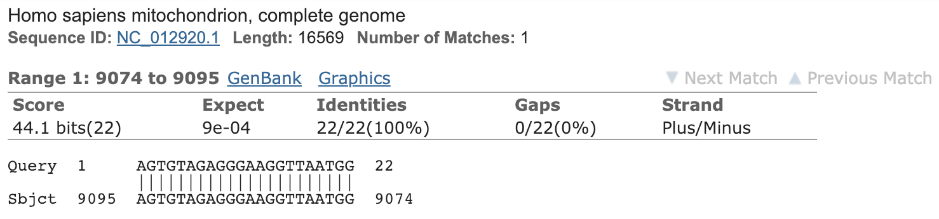


## Comparison of MitoScape Benchmark MtDNA sequences to validation sequences in mtDNA-Server.

There are a number of improvements in the Benchmark mtDNA set that we created versus the validation set used in mtDNA-Server(Weissensteiner et al. 2016). First, the mtDNA-Server set comprised two cancer samples versus nine samples from PBMCs in MitoScape. In both validation sets, mtDNA-enriched data was obtained by long-range PCR amplification. But, in the case of MitoScape, we validated and rigorously tested our PCR primers for amplification of NUMTs. We found no evidence of testing or validation in the mtDNA-Server set. Furthermore, since the vast majority of current WGS studies contain samples from PBMCs, using PBMCs is a more appropriate validation set for WGS data. Second, we used 2x300bp paired-end reads for sequencing of our mtDNA-enriched compared to the 2x101bp paired-end reads used in mtDNA-server. Reads from our data are three times longer and give more accurate alignments, which is critical in avoiding false alignments of NUMTs to mtDNA. The shorter reads used in mtDNA-server are shorter than many of the known NUMTs. Third, the mixing experiments used to create heteroplasmy values in mtDNA-Server are unlikely to produce reliable heteroplasmy estimates. We found no evidence of validation of the heteroplasmy estimates in mtDNA-Server, similar to the measurements of heteroplasmy that we performed. Thus, we concluded that the validation set used in mtDNA-server was inadequate, and required us to create our own Benchmark set for MitoScape.

**Penn Medicine Biobank**

The Penn Medicine Biobank (PMBB) is an academic biobank that provides researchers with centralized access to a large number of blood and tissue samples with attached health information. Participants actively consent to allow the linkage of biospecimens to their longitudinal electronic health record (EHR). Currently, > 50,000 participants are enrolled in PMBB and ≈23% of these individuals self-identify as having African Ancestry. This study included a subset of 10,289 individuals who have undergone who exome sequencing, performed through a collaboration with the Regeneron Genetics Center. Sample preparation and exome sequencing were completed per the standard Regeneron Genetics Center methodology, as previously described(Dewey et al. 2016). The characteristics of the cohort are described in S1 Table.

## References

Dewey FE, Gusarova V, O’Dushlaine C, Gottesman O, Trejos J, Hunt C, Van Hout CV, Habegger L, Buckler D, Lai K-MV, et al. 2016. Inactivating Variants in ANGPTL4 and Risk of Coronary Artery Disease. *New England Journal of Medicine* **374**: 1123–1133.

Weissensteiner H, Forer L, Fuchsberger C, Schöpf B, Kloss-Brandstätter A, Specht G, Kronenberg F, Schönherr S. 2016. mtDNA-Server: next-generation sequencing data analysis of human mitochondrial DNA in the cloud. *Nucleic Acids Res* **44**: W64–W69.
