# Supplementary figures and images for: MitoScape: A big-data, machine-learning platform for obtaining mitochondrial DNA from next-generation sequencing data
Source: PLoS Comput Biol. 2021 Nov 11;17(11):e1009594. doi: 10.1371/journal.pcbi.1009594 (PMC8610268; doi:10.1371/journal.pcbi.1009594)

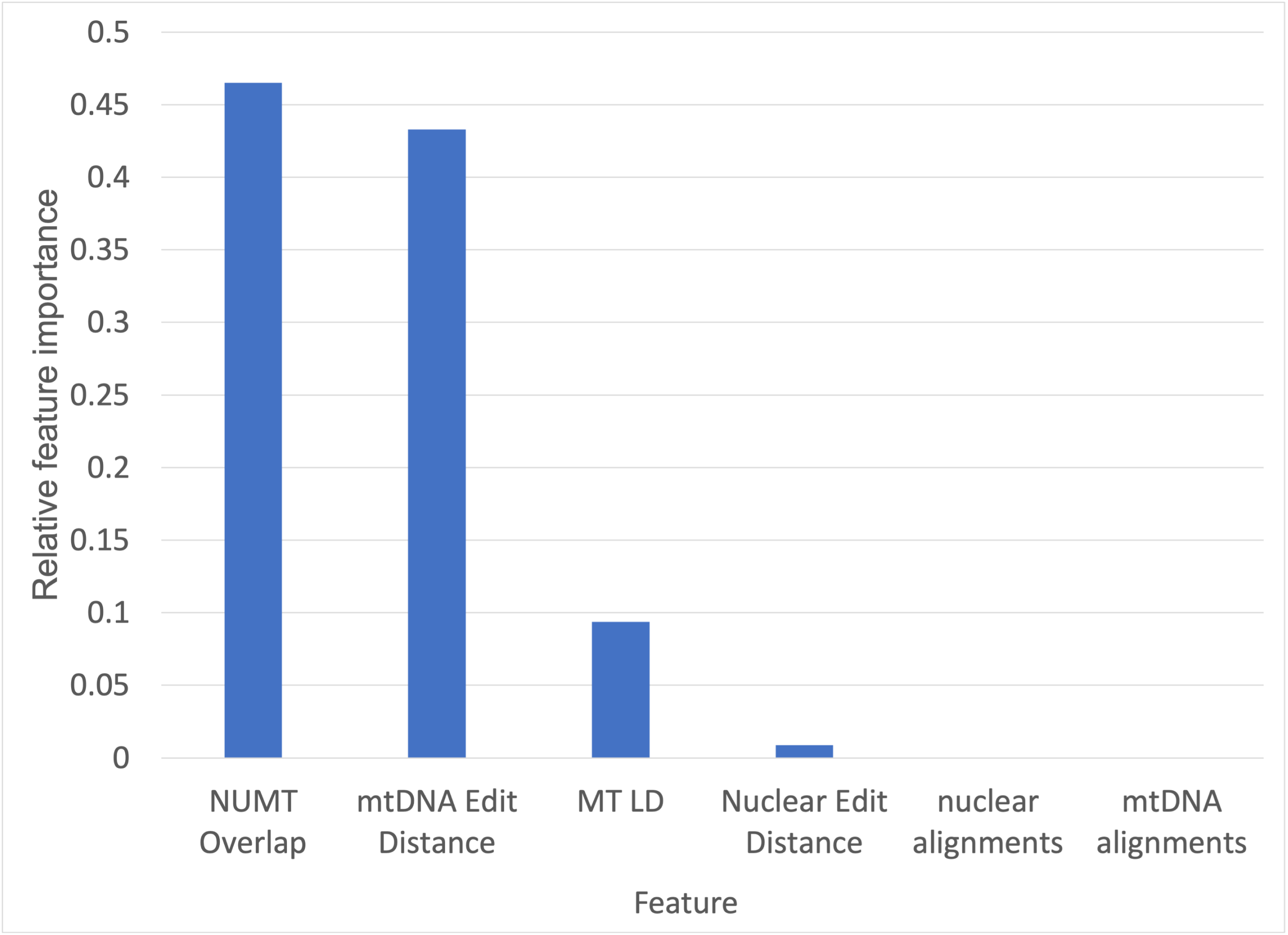

Supplement: S1 Fig — Plot of relative feature (variable) importance scores (y-axis) after training of random forest classifier in MitoScape. Each feature is displayed on the x-axis. Feature importance scores of all variables sums to one, and the higher the relative variable importance score, the more important this feature was in the classification procedure. (TIF) [file pcbi.1009594.s002.tif]

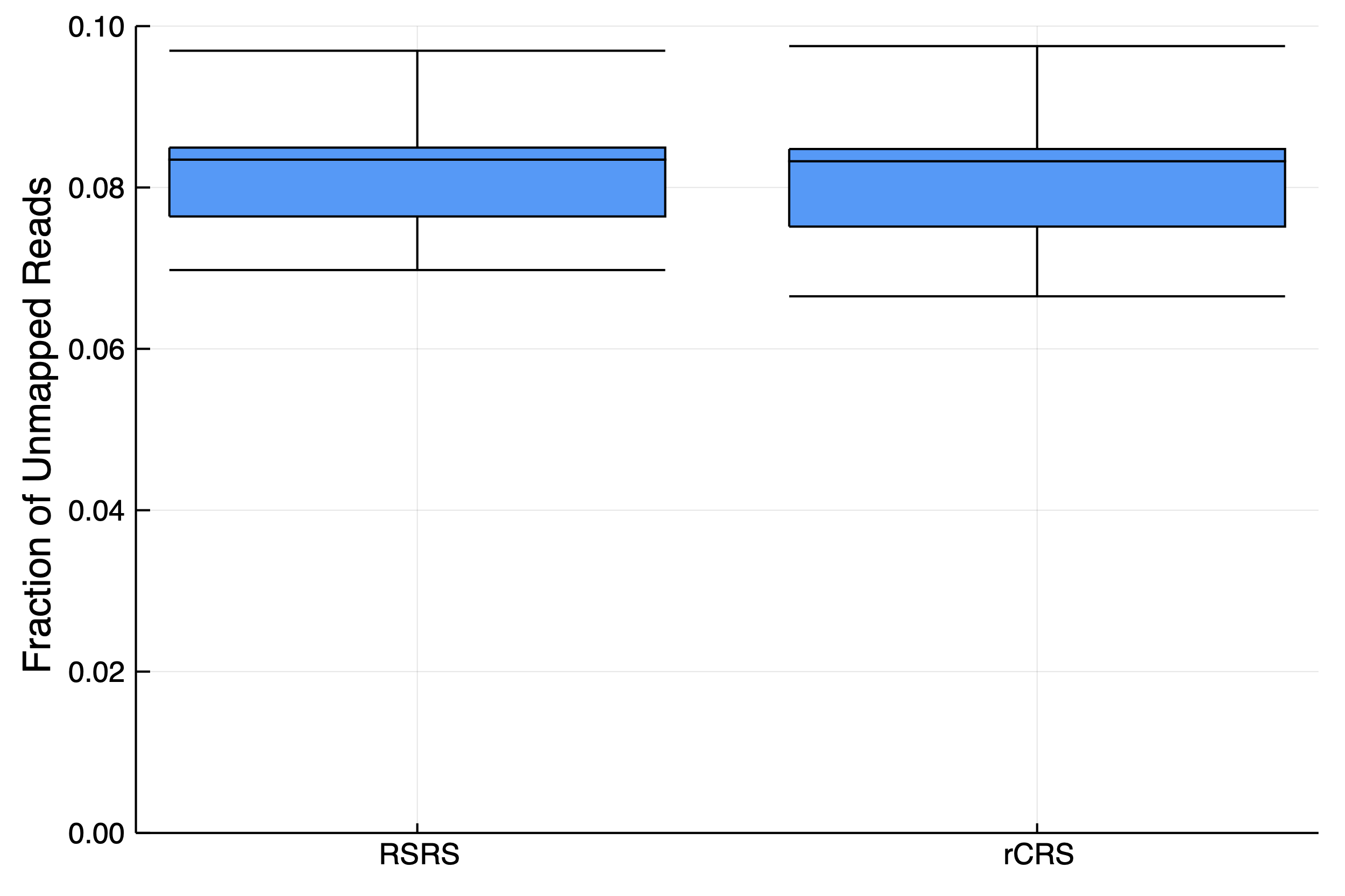

Supplement: S2 Fig — (TIF) [file pcbi.1009594.s003.tif]

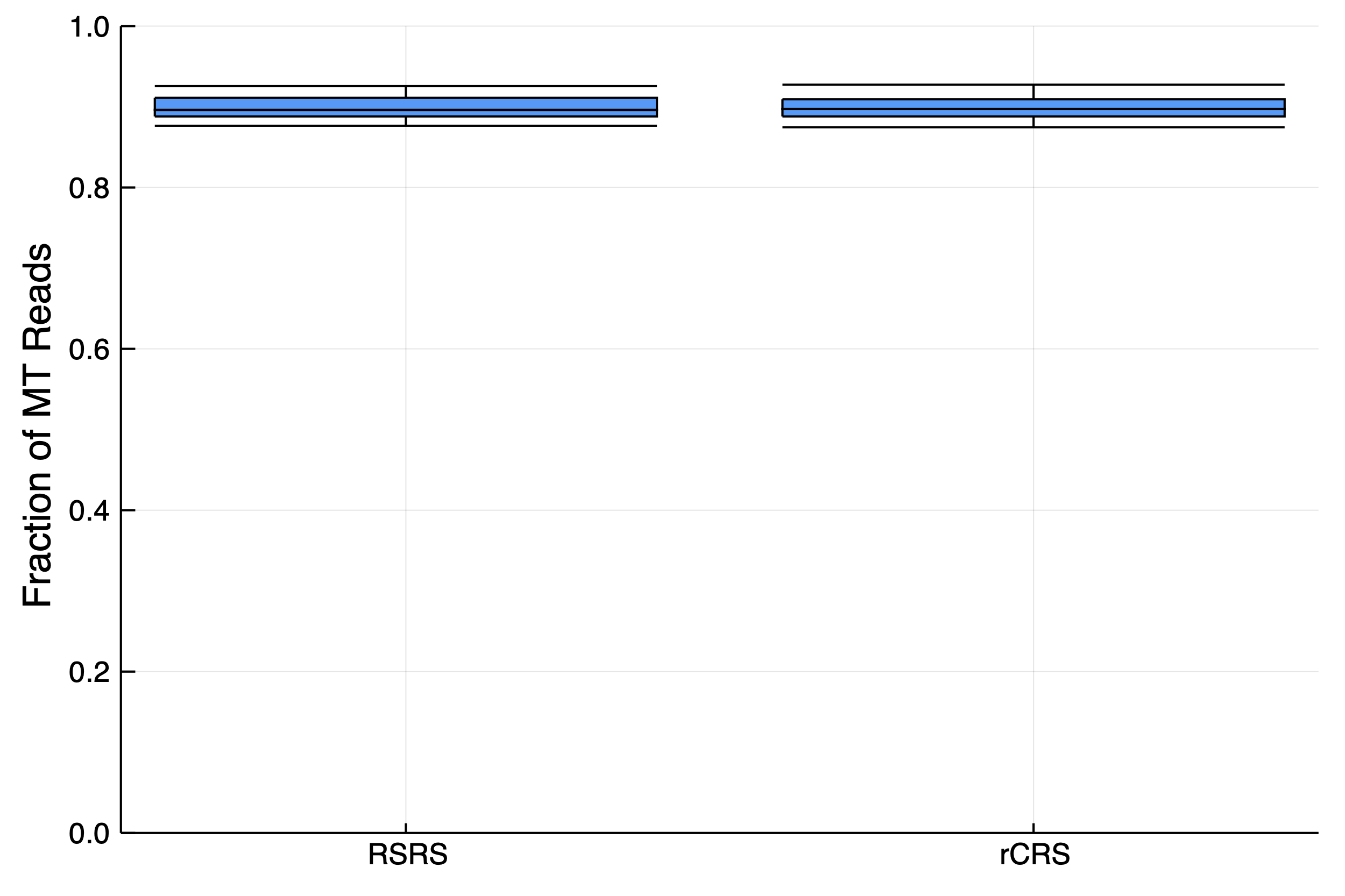

Supplement: S3 Fig — (TIF) [file pcbi.1009594.s004.tif]

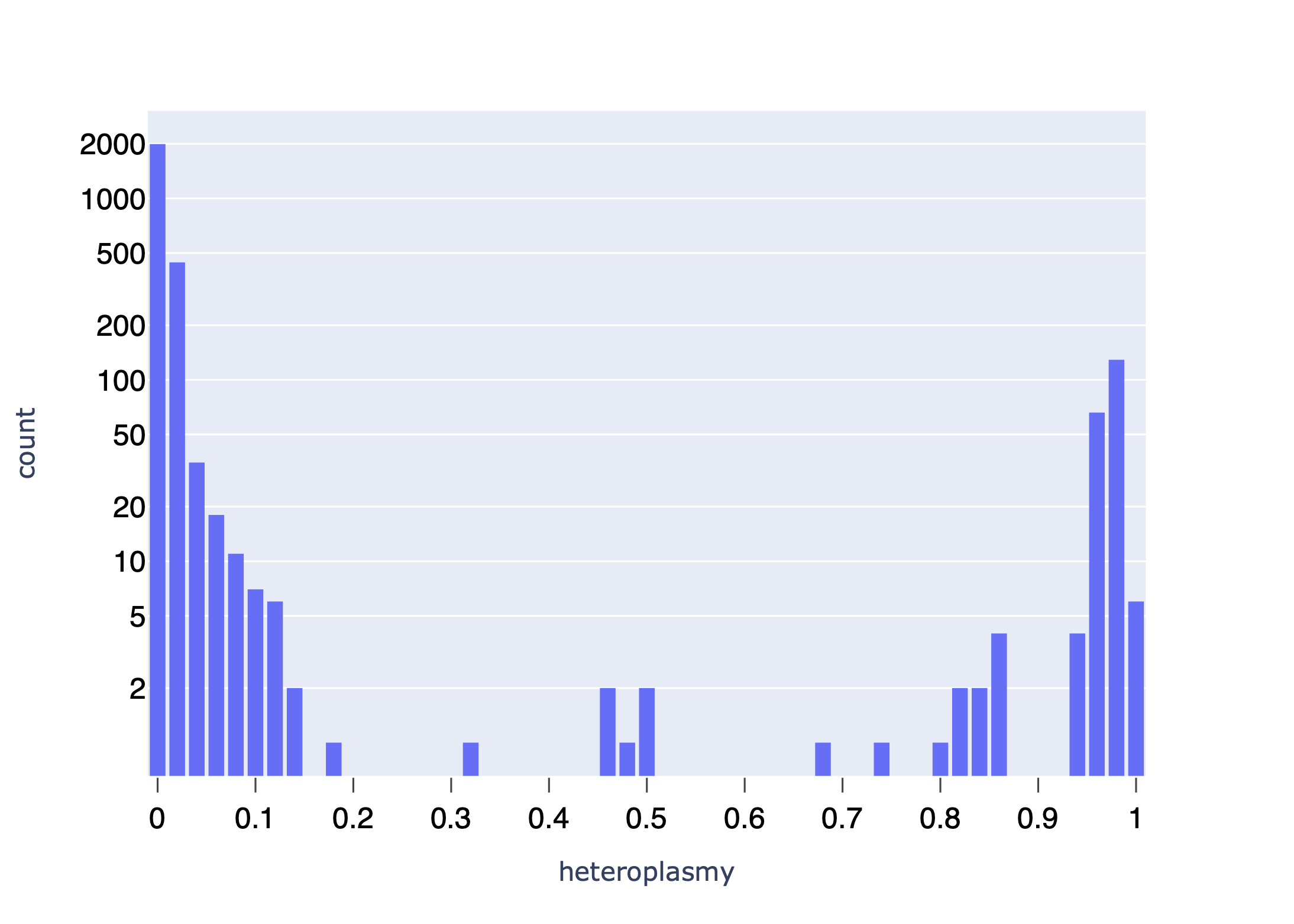

Supplement: S4 Fig — (TIF) [file pcbi.1009594.s005.tif]

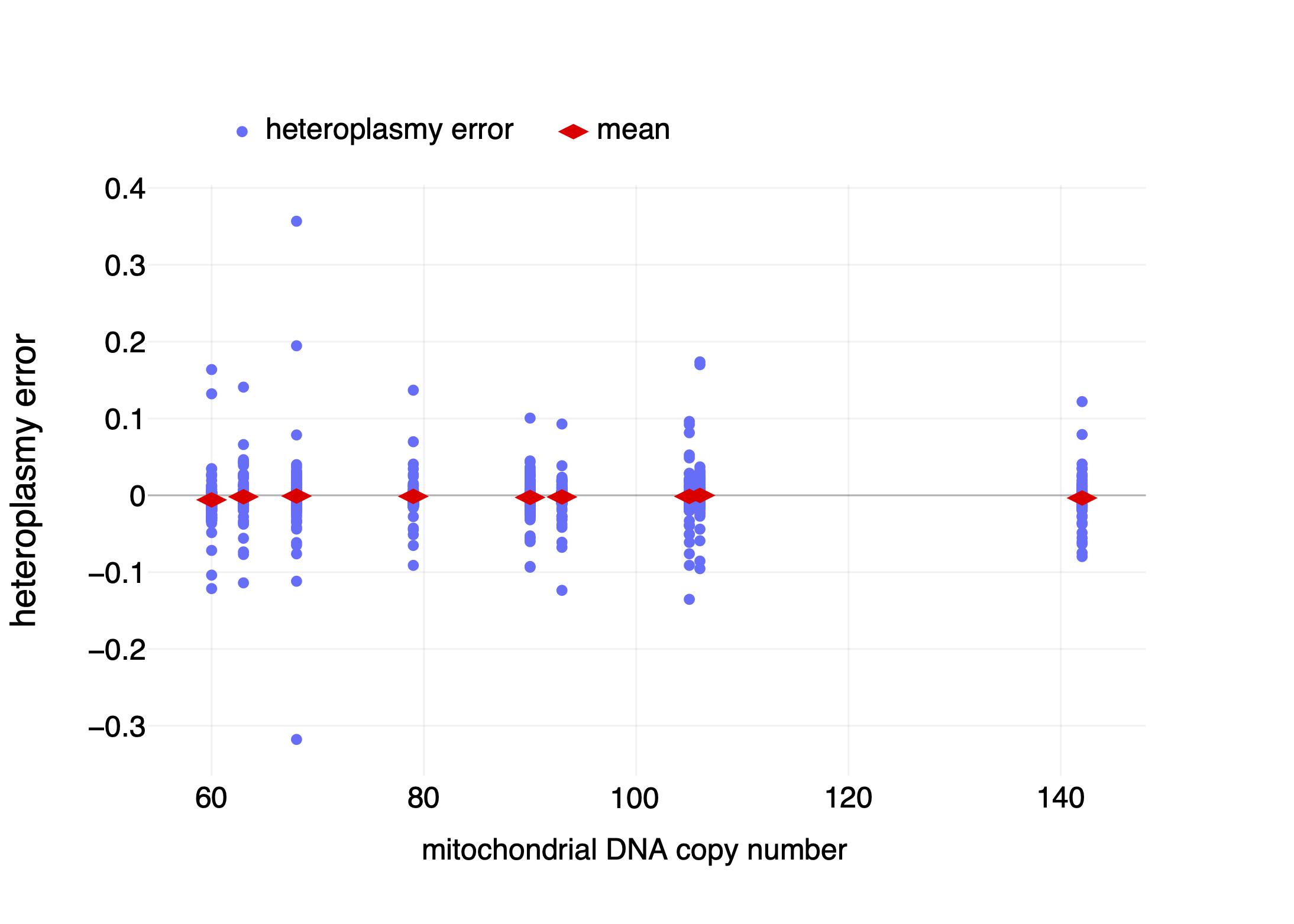

Supplement: S5 Fig — Each blue circle represents the heteroplasmy error and mitochondrial copy number for a single variant. (TIF) [file pcbi.1009594.s006.tif]

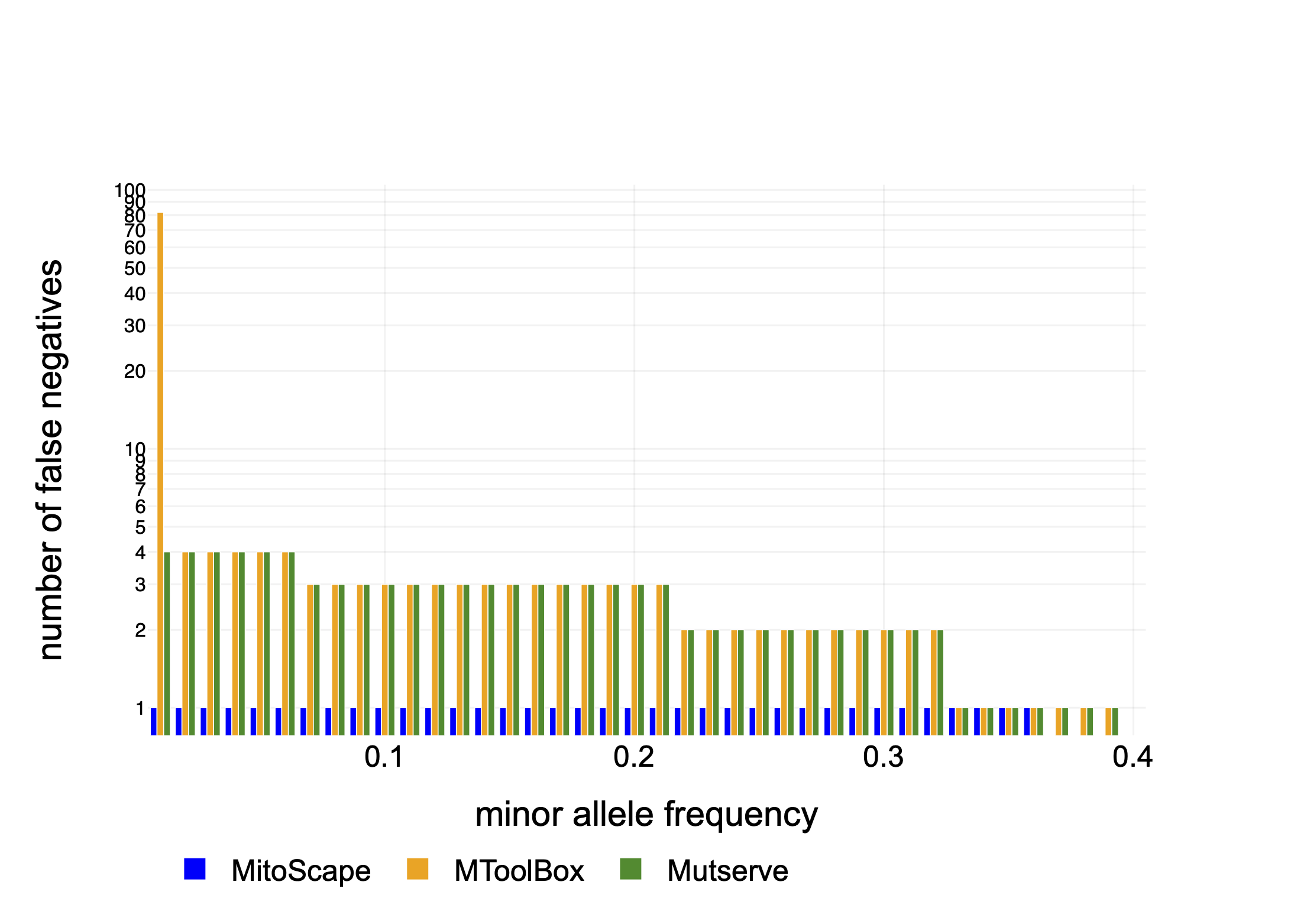

Supplement: S6 Fig — The y-axis represents the cumulative number of false negatives where the corresponding actual heteroplasmy is less than the value on the x-axis. The x-axis represents minor allele frequency, and therefore, is between 0 and 0.5. Minor allele frequency is equal to actual heteroplasmy if actual heteroplasmy is < 0.5, and equal to 1-actual heteroplasmy, otherwise. (TIF) [file pcbi.1009594.s007.tif]
