# Supplementary material for: MitoScape: A big-data, machine-learning platform for obtaining mitochondrial DNA from next-generation sequencing data
Source: PLoS Comput Biol. 2021 Nov 11;17(11):e1009594. doi: 10.1371/journal.pcbi.1009594 (PMC8610268; doi:10.1371/journal.pcbi.1009594)
Supplement: S1 Table — (DOCX) [file pcbi.1009594.s008.docx]

**S1 Table: Penn Medicine Biobank Participant Characteristics**

|  | **PMBB** |
| --- | --- |
| Total Patients | 10283 |
| Male (%) | 6091 (59.2%) |
| Age (SD) | 69.3 (14.1) |
| **Race** |  |
| Black or African American | 2031 |
| Other | 862 |
| White | 7390 |
