# Supplementary material for: MitoScape: A big-data, machine-learning platform for obtaining mitochondrial DNA from next-generation sequencing data
Source: PLoS Comput Biol. 2021 Nov 11;17(11):e1009594. doi: 10.1371/journal.pcbi.1009594 (PMC8610268; doi:10.1371/journal.pcbi.1009594)
Supplement: S2 Table — (DOCX) [file pcbi.1009594.s009.docx]

**S2 Table**: Haplogroup demographics of subjects used in hypertrophic cardiomyopathy-mitochondrial haplogroup association from Penn Biobank data.

| **Haplogroup** | **Total** | **Number of Men** | **Percent Men** | **Mean Age** | **SD Age** |
| --- | --- | --- | --- | --- | --- |
| N' | 466 | 289 | 62.02 | 70.10 | 13.00 |
| M | 45 | 22 | 48.89 | 66.34 | 15.06 |
| X | 113 | 67 | 59.29 | 71.95 | 14.67 |
| R0 | 3,529 | 2,182 | 61.83 | 70.31 | 13.61 |
| T | 696 | 424 | 60.92 | 69.50 | 13.68 |
| U | 1,676 | 1,026 | 61.22 | 70.07 | 13.46 |
| J | 659 | 410 | 62.22 | 68.97 | 13.73 |
| **Total** | 7,184 | 4,420 | 61.53 | 70.00 | 13.60 |
