# Supplementary material for: MitoScape: A big-data, machine-learning platform for obtaining mitochondrial DNA from next-generation sequencing data
Source: PLoS Comput Biol. 2021 Nov 11;17(11):e1009594. doi: 10.1371/journal.pcbi.1009594 (PMC8610268; doi:10.1371/journal.pcbi.1009594)
Supplement: S3 Table — Reference haplogroup is R0. Adjustment for multiple testing was done by Bonferroni correction. Logistic regression was performed using R. (DOCX) [file pcbi.1009594.s010.docx]

**S3 Table**: Logistic regression analysis with HCM as dependent variable and mitochondrial haplogroups, age, and the first five principal components of the nuclear genetic variants PCA analysis as covariates, for men only. Reference haplogroup is R0. Adjustment for multiple testing was done by Bonferroni correction. Logistic regression was performed using R.

| Covariate | beta | Std error | p-value | Adj. p-value | OR | 95% Conf. Interval | Case | Control |
| --- | --- | --- | --- | --- | --- | --- | --- | --- |
| U | 0.68 | 0.33 | 0.040 | 0.240 | 1.98 | [1.03, 3.80] | 18 | 1008 |
| T | 1.26 | 0.37 | 5.752e-4 | 0.003 | 3.52 | [1.72, 7.21] | 13 | 411 |
| J | 0.89 | 0.41 | 0.031 | 0.185 | 2.42 | [1.09, 5.41] | 9 | 401 |
| N' | -0.23 | 0.75 | 0.762 | 1.000 | 0.80 | [0.18, 3.45] | 2 | 287 |
| M | -13.80 | 1373.00 | 0.992 | 1.000 | 1.02e-06 | [0.00, Inf] | 0 | 22 |
| X | -13.73 | 783.10 | 0.986 | 1.000 | 1.08e-06 | [0.00, Inf] | 0 | 67 |
| Age | -0.04 | 0.01 | 8.456e-5 | 8.456e-5 | 0.96 | [0.95, 0.98] |  |  |
| PC1 | -130.42 | 315.60 | 0.679 | 0.679 | 2.28e-57 | [0.00, 9.54e211] |  |  |
| PC2 | -335.76 | 274.40 | 0.221 | 0.221 | 1.52e-146 | [0.00, 6.05e87] |  |  |
| PC3 | -43.60 | 40.21 | 0.278 | 0.278 | 1.16e-19 | [6.95e-54, 1.95e15] |  |  |
| PC4 | 92.95 | 102.30 | 0.364 | 0.364 | 2.34e40 | [1.89e-47, 2.89e127] |  |  |
| PC5 | 6.35 | 107.40 | 0.953 | 0.953 | 570.80 | [2.387e-89, 1.37e94] |  |  |
