# Supplementary material for: MitoScape: A big-data, machine-learning platform for obtaining mitochondrial DNA from next-generation sequencing data
Source: PLoS Comput Biol. 2021 Nov 11;17(11):e1009594. doi: 10.1371/journal.pcbi.1009594 (PMC8610268; doi:10.1371/journal.pcbi.1009594)
Supplement: Attachment — Submitted filename: ResponseToReviewers.docx [file pcbi.1009594.s011.docx]

Dear Reviewers,

We thank you for your insightful and detailed comments. We have modified the manuscript based on your valuable comments. We have provided two versions of the manuscript: one clean, without tracking, version and one version with changes tracked. Below we have provided our responses below to each of your comments (reviewers’ comments are outlined in red font, our responses are in black font). We thank you again for your time and thoughtful comments.

Yours respectfully on behalf of all the authors,

Larry N. Singh, Ph.D.

Reviewer #1: Comments on “MitoScape: A big-data, machine-learning platform for obtaining mitochondrial DNA from next-generation sequencing data”

I only have one minor suggestion.
An important factor influencing mtDNA variant detection is mtDNA copy number. Normally, samples with lower mtDNA copy numbers would be more significantly affected by reads from NUMTS. I wonder what distribution of mtDNA copy numbers in the nine testing blood samples is, given the corresponding data were generated by WGS. Does mtDNA copy number affect performance of mtDNA variant identification, especially identification of heteroplasmies? Providing copy number data could also illustrate another important use of MitoScape, as mentioned in Discussion.

We thank the reviewer for the excellent observation and thoughtful hypothesis. We did indeed test this hypothesis (page 10, Supplementary Figure S5) using the common method of computing mtDNA copy number from NGS based on the relative ratio of mtDNA to nDNA reads. We found no apparent correlation between mtDNA copy number and heteroplasmy error, however. We speculate that the reason for this lack of correlation is the small N (9 samples), and the low variation in mtDNA copy number (mean=90, standard deviation=25). We would likely need a broader range of samples to test this hypothesis. We have updated the manuscript on page 10 to include these findings.

Reviewer #2: The accurate identification of mitochondrial DNA (mtDNA) variants, especially low-frequency heteroplasmies, in sequencing data is an important problem in mitochondrial genetics. This problem is especially acute due to the presence of nuclear DNA fragments masquerading as mitochondrial DNA in sequencing data, commonly known as “NUMTs”. There are many approaches available to minimize NUMT contamination in sequencing data. However, these are largely heuristic, rely on the use of arbitrary filters, and there is often no clear measure of their efficacy/accuracy. Singh et al. provide an effective, scalable method to deal with these problems. I have a number of questions and comments to which I would like to hear the authors’ response:

The authors mentioned 10 samples enriched for mtDNA sequences as positive control. Am I correct in thinking that these independent from the 9 samples used as the test set? Perhaps make this clearer in the main text.

Yes, the reviewer is correct, the 9 test samples using for testing are completely different from the 10 samples used as positive control. We have followed the reviewer’s suggestion and clarified this point in the main text (page 8).

They mention using k-fold cross-validation with 80% of the training set and 20% as validation. Could the authors describe this in more detail? There are 8 samples in the negative training set and 10 in the positive set. How were they split? Or was the separation into training and validation sets carried out on the read level? How many iterations of the k-fold validation were carried out? Could the authors provide performance metrics from the k-fold validations? Can they also provide the weights that the classifier assigns to each of the features used? Additionally, can they also generate a plot of the probability of correctly assigning a read to mt/NUMT sequences as a function of these features in their validation? These metrics will be useful in understanding the classifier and how important each is in discriminating mitochondrial and NUMT sequences.

The reviewer is indeed right that the separation of k groups was performed at the read level. All reads from all samples were combined and then randomly split into 80% (4 groups) for training and 20% (1 group) for validation. We have clarified this point in the main text (page 6).

An 80%-20% split implies 5-fold cross-validation, i.e. k=5, by definition. In k-fold cross validation, for each of the k groups, one is chosen as a test group, and the other k-1 as training groups, so by definition there are k iterations. We reported the standard F1 score measurement of accuracy of the training procedure in the text (page 6).

The random forest (RF) method is an ensemble method and is somewhat of a “black box” approach. Hence, getting weights for each feature is not possible, and interpreting the resulting model is difficult. Instead, we use the standard “variable importance” or “feature importance” measures to indicate the “weight” of each feature. We have plotted the relative feature importance scores normalized to sum to 1.0 in Supplementary Figure S1. The higher the relative importance score for the feature, the more important is that feature in the classification procedure.

I am not sure using the absolute difference in heteroplasmy frequency between the benchmark and test set is the appropriate way to measure the performance of the method. Higher frequency heteroplasmies are more likely to vary in frequency just by sampling error. Perhaps more accurate would be to scale the difference by p(1-p) where p is the heteroplasmy fraction in the benchmark dataset. It would also be helpful to see this difference with respect to the frequency of the heteroplasmy. Please also provide similar metrics or a figure (e.g. as in Fig 3) for the other methods for a direct comparison with Mitoscape.

The reviewer raises an excellent point, and hence, we adopted the reviewer’s suggestions in our new Figures. We have added Figures 3B and 4B which report the scaled heteroplasmy levels. As the reviewer will note, MitoScape clearly outperforms the other two software when using this new scaled heteroplasmy error.

I noticed in Fig. 4 that Mutserve matches or performs better than Mitoscape at higher sequencing depth (>1200). Would this be a fair assessment? If so, perhaps mention this as it does not take away from the fact that Mitoscape performs well at lower sequencing depths.

The reviewer makes an interesting observation and we have made note of this observation in the manuscript (pages 10 and 12). We also add that the difference among the three tools for read depths greater than 1200 is negligible.

I do think an absolute difference of 0.2 is appropriate to define false negative or positive heteroplasmies. One reason for this is that a difference of 0.2 matters means different things at low and high frequency heteroplasmies. For example, at a frequency of 0.2, a difference of -0.2 genuinely indicates a failure to detect the heteroplasmy whereas the same difference at a frequency of 0.5 does not. Additionally, I feel that heteroplasmy classification errors (as defined here) should be measured by detection (or failure of) heteroplasmies, and not based on difference in frequency of known heteroplasmies. For example, a false positive could be detection of a heteroplasmy above some frequency threshold (e.g. 0.05) which did not exist in the benchmark data.

The actual difference in heteroplasmy error is important in diagnosing mitochondrial disease. We have written extensively on this topic and recently provided the guidelines for the ACMG/AMP standards for mtDNA variant interpretation (see: PMCID: PMC7717623, which we have also now cited). For many diagnoses, knowing accurate heteroplasmy levels for mtDNA variants is critical. Nevertheless, we absolutely agree with the reviewer that detection error is another excellent and enlightening means of measuring the performance of the different tools. Hence, we also added detection error (fraction of detected heteroplasmic variants) as a performance measure to our manuscript (Figure 5 and page 12).

I did not understand the purpose of the section “Application to complex human disease: hypertrophic cardiomyopathy”. Was Mitoscape used to call mtDNA reads for haplogroup calling? How much more accurate was this process compared to just calling haplogroups from the sequence data (without any filters) using Haplogrep (for example)? Alternatively, how much more accurate was Mitoscape compared to other methods described in the paper? In other words, I see the benefit of using Mitoscape to call low-frequency heteroplasmies, but I don’t see how beneficial it is for haplogroup calling.

As shown in Table 2, both MToolBox and Mutserve produced many homoplasmic variant errors, whereas MitoScape did not produce any homoplasmic variant errors. Since accurate homoplasmic mtDNA variant calls are required for haplogroup computation, both MToolBox and Mutserve could potentially lead to inaccurate haplogroup computation. Unfortunately, we do not have access to the samples used for any of these analyses, so there was no way to test the accuracy of the haplogroup calls. This section was meant to show how MitoScape might be used in a real-world application. We have added text (page 13, Applications section) to clarify this point; however, we are also amenable to moving this section to the Supplementary text.

Please provide details of the number and frequency distribution of heteroplasmies identified in the 9 test samples.

We thank the reviewer for this improvement suggestion; we have now added Supplementary Figure S4 which summarizes these statistics.
